# Know Thyself! Predicting Subjective Well-Being from personality estimation discrepancy and self-insight

**DOI:** 10.1007/s12144-022-03396-1

**Published:** 2022-08-04

**Authors:** August Håkan Nilsson, Kira Friedrichs, Petri Kajonius

**Affiliations:** Department of Psychology, Lund University, Lund, Sweden

**Keywords:** Personality estimation discrepancy, Subjective well-being, Big five, Self-discrepancy, Self-insight

## Abstract

Discrepancies in views of the Self are suggested to be negatively related to well-being (Higgins, 1987). In the present study, we used a novel concept, Personality Estimation Discrepancy (PED), to test this classic idea. PED is defined as the computed difference between how one view oneself (Self-Perceived Personality) and a standard Big Five test (IPIP-NEO-30). In a pre-registered (osf.io) UK online study (*N* = 297; M_age_ = 37, SD = 14) we analyzed: (1) whether PED would predict Subjective Well-Being (SWB; Harmony in Life, Satisfaction with Life, Positive affect, Negative Affect) and Self-Insight, and (2) whether Self-Insight would mediate the relationship between PED and SWB. The results showed that underestimation of Extraversion, Conscientiousness, and Emotional Stability indeed is associated with both high SWB and high Self-Insight. However, these effects mostly disappeared when controlling for the Big Five test scores. Furthermore, Self-Insight largely (42.9%) mediated the relationship between the mis-estimation and SWB. We interpret these finding such that the relationship of mis-estimating one’s personality with SWB and Self-Insight are mostly explained by the Big Five factors, yet the discrepancy is a dependent feature of scoring particularly high or low on certain personality traits.

**Supplementary Information:**

The online version contains supplementary material available at 10.1007/s12144-022-03396-1.

For most people, well-being is one of the greatest goals in life (Diener et al., 1998; Roberts & Robins, 2000) and is associated with various positive outcomes such as physical, emotional, and social health (Ngamaba et al., 2017), as well as academic achievement (Buecker et al., 2020). Among the strongest predictors of SWB are the Big Five personality traits (Anglim et al., 2020). One aspect of personality that has been shown to predict well-being is Self-Discrepancy, i.e., disagreement between the ideal/ought self and the actual self (see Self-Discrepancy Theory; Higgins 1987; Kelly et al., 2015; McDaniel & Grice, 2008; Pavot et al., 1997). This classic idea led us to explore one novel aspect of Self-Discrepancy and Personality that to our knowledge has not yet been investigated – Personality Estimation Discrepancy (PED). In the present study, we operationalize this as the computed difference in how individuals perceive their own personality and their personality in terms of a Big Five test score.

Over time, popular non-scientific personality tests such as the 16 personalities (https://www.16personalities.com/*)* or the four colors of personality (Erikson, 2019) have become particularly common in the general population. This may further have caused many individuals to make erroneous estimations about their personality, which arguably makes it relevant to investigate how PED relates to well-being. In this same vein, Self-Insight, the ability to understand one’s feelings, thoughts, and behaviors, is another strong predictor of SWB (Lyke, 2009; Stein & Grant, 2014), which strengthens our idea of the relevance of exploring PED. A high level of Self-Insight would be analogous to a low PED.

The aim of this paper is to explore and test how PED relates to SWB and Self-Insight. This is important because it links Personality, Self-Insight, and Self-Discrepancies as predictors of SWB (a highly valued outcome for individuals and society).

## Subjective Well-Being (SWB)

SWB is mostly defined as the subjective evaluation of an individual’s life (Diener, 2000). This is constituted by a cognitive component, most commonly seen as Satisfaction with Life (Diener et al., 1985), and an affective component, measured as Positive Affect over Negative Affect (Watson et al., 1988). The cognitive component Satisfaction with Life has been proposed to be missing important aspects of well-being such as relationships, inner harmony, and balance (Delle Fave et al., 2016). For example, when individuals reported what happiness is to them around the globe, the most common answer was Harmony, twice as common as Satisfaction (Delle Fave et al., 2016). Consequently, more light has been shed on Harmony in Life (Kjell et al., 2016). These four constructs seem to capture most aspects of SWB (Kjell et al., 2016; Watson et al., 1998). Whereas Personality and Self-Discrepancies have been extensively examined in relation to the classical SWB approach (Anglim et al., 2020; Pavot et al., 1997), they have not been examined with Harmony. We consider Harmony in Life, as well as the three classical mentioned constructs in this study, using a composite of them.

## Personality Estimation Discrepancy (PED)

The most widely known personality model is Costa and McCrae’s Five-Factor Model (1987) and the Big Five personality traits. These are in brief; Extraversion, how talkative and outgoing someone is; Emotional Stability (the lower end of the Neuroticism spectrum), how calm and stable someone is; Conscientiousness, how tidy and detailed someone is; Agreeableness, how trusting and forgiving someone is; and Openness, how reflecting and imaginative someone is (for a more detailed description, see McCrae & Costa 2008). These traits are strongly related to SWB (Anglim et al., 2020).

Looking beyond the Big Five, research in the fields of social and personality psychology has produced a plethora of studies concerning self-*discrepancies*. The greatest share is focused on Higgins’ Self-Discrepancy Theory (Higgins, 1987; Higgins, 1989; Mason et al., 2019; Mcdaniel & Grice, 2005; McDaniel & Grice, 2008). Self-Discrepancy Theory states that individuals have three different internalized self-state representations, which are the Actual Self, how one really is, the Ideal Self, how one wishes to be, and the Ought Self, how one feels one should be. Specifically, according to Higgins’ Theory, discrepancies between the Actual Self and the Ideal/Ought Self increase emotional vulnerability (Higgins, 1987; 1989). Research has demonstrated that discrepancies are negatively related to SWB (Pavot et al., 1997) and predict an increase in factors related to psychological well-being such as anxiety, self-esteem, and depression (Kelly et al., 2015; McDaniel & Grice, 2005, 2008).

In the Self-Discrepancy research, individuals are asked about their own understanding of their different self-states on a general level (Higgins, 1987). Not much research has specified the self-states in the form of personality traits and created personality trait Self-Discrepancies (with a few exceptions, see McDaniel & Grice 2005). Nor has much research operationalized the self-representations of the Actual Self indirectly (i.e., not asking explicitly what a person’s Actual/Ideal/Ought self is), which is how personality is generally assessed. One exception is a study by McDaniel & Grice (2005) in which they operationalized the Actual Self from a Big Five inventory. However, they did not access the self-perceived self, i.e., asking individuals explicitly about their trait scores without measuring them with an inventory designed to, as objectively as possible, measure personality. We explore this by computing the difference between first, the personality derived from a self-estimation of one’s personality traits. In terms of the Self-Discrepancy theory, this can be seen as the *explicit* Actual Self in personality traits. It is explicit because it directly asks individuals about their levels of personality traits. We refer to this measure as the Self-Perceived Personality since it reflects individuals’ direct subjective understanding of their personality traits. And second, the personality derived from a scientifically founded personality test (IPIP-NEO-30, Kajonius & Johnson 2019). In terms of the Self-Discrepancy theory, this can be seen as the *implicit* Actual Self in personality traits. It is implicit because it does not explicitly ask individuals about their estimation of their personality traits. For clarity, we refer to this measure as the Actual personality, since it is the generally accepted operationalization of measuring personality traits in the literature.

The absolute discrepancy between these measures (absolute PED) can be seen as the general discrepancy between the (implicit) Actual personality and the Self-Perceived personality. The directed discrepancy between them (directed PED) includes both personality overestimation (when the Self-Perceived personality score is higher than the score in Actual personality) and personality underestimation (when the Self-Perceived personality score is lower than the scientifically founded personality test reflects). Both the absolute and the directed PED can be described as mis-estimations of one’s personality. Besides Self-Discrepancy Theory, the directed PED may further contribute to the self-enhancement literature. In contrast to the Self-Discrepancy theory, research has shown that individuals who overestimate various traits and their SWB report higher levels of SWB (Dufner et al., 2019; Wojcik & Ditto, 2014). Considering the positive association between SWB and the Big Five traits, particularly Extraversion, Emotional Stability, and Conscientiousness (Anglim et al., 2020), directed PED in these traits may be positively associated with SWB.

## Self-insight

We describe PED as a mis-estimation of one’s personality, thus it should be related to Self-Insight, the ability to understand one’s feelings, thoughts, and behaviors. Self-Insight, just like Self-Discrepancy, has typically been strongly related to SWB (Harrington et al., 2014, 2016; Harrington & Loffredo, 2010; Lyke, 2009; Silvia & Phillips, 2011; Stein & Grant, 2014). According to our theoretical framework, a high PED would relate to low Self-Insight. Consequently, a person high on Self-Insight would, if their understanding of the traits is accurate, rate themselves (Perceived Self) rather congruent with their scale scores (Actual Self). We further propose that Self-Insight (partly) mediates the relationship between PED and SWB. Self-insight mediates the relationship between Self-Reflection and SWB (Stein & Grant, 2014) and we propose a similar mechanism for PED. Since personality traits are stable from early life (Costa & McCrae, 2008), Self-Insight would partly stem from accurate estimations of one’s personality traits that may develop throughout life.

## The Present Study

The present study aims to investigate whether computed PED is negatively related to SWB and Self-Insight, based on Higgin’s (1987) classic Self-Discrepancy Theory.

### Hypothesis

1a. PED is negatively related to SWB.

### Hypothesis

1b. PED is negatively related to Self-Insight.

### Hypothesis 2

Self-Insight mediates the relationship between PED and SWB^1^.

## Method

### Participants and Procedure

According to pre-registration, 300 UK adult participants were recruited from *Prolific* (https://www.prolific.co/), a website platform for scientists to recruit participants, including full-time workers, as well as unemployed and students (for Prolific’s use in academia, see Palan & Schitter 2018). Three participants did not answer all questions and were thus, as conditioned in the pre-registration, excluded from further analysis. Out of the 297 participants, 202 were female and 95 were male. The mean age was 37 (*SD* = 14; Min-Max = 18–76) and 158 had at least a bachelor’s degree.

Participants were provided with a link for the online questionnaire on Prolific. After being briefed about the study and agreeing to the privacy protection conditions, participants were provided with the scales and items described in the instrument section and were at completion debriefed about the study. Answering the questionnaire took on average 9.65 (*SD* = 4.69) minutes and participants received monetary compensation of 0.9£. All data were collected between 4 pm and 6 pm, the 5th of May 2021.

### Instruments

**Self-Perceived Personality.** To examine Self-Perceived Personality, participants were given descriptions of each of the Big Five traits, one at a time, and then rated these numerically on a scale (1-100). The descriptions were inspired by the Single-Item Measures of Personality (Woods & Hampson, 2005) and followed by the explicit rating question: “In general, how extroverted/neurotic/agreeable/conscientious/open are you?”. Neuroticism was reversed to reflect Emotional Stability.

**Personality Measurement (IPIP-NEO-30)**. The International Personality Item Pool 30 item version (IPIP-NEO-30, Kajonius & Johnson 2019) was used. This scale includes the 30 items from the IPIP-NEO-120 (Johnson, 2014), with six items for each of the Big Five traits (e.g., “Have a lot of fun” for Extraversion, or “Carry out my plans” for Conscientiousness) and were answered on Likert scales ranging from 1 to 5 (*Very inaccurate* to *Very accurate*) on how much one agrees with the statement. Due to the ongoing Covid-19 pandemic, the Extraversion item “Avoid crowds” was substituted for “Love large parties”. All Cronbach’s alphas were over 0.75 and all McDonald’s omegas were at least 0.82.

**Personality Estimation Discrepancy (PED).** The main independent variable in the present study was the PED. This was constructed for each Big Five trait by subtracting the standardized Big Five scores (IPIP-NEO-30) from the standardized Self-Perceived Personality scores. The first absolute PED variable did not consider the direction (minus or plus) yielding only the size of the discrepancy. High values in the PED indicated a high discrepancy, no matter if the participants over-or underestimated a trait. The second relative *directed PED* variable considered the direction of estimates, e.g., over-or underestimation of a trait. A positive score in directed PED indicated overestimation (i.e., higher Self-Perceived Personality than Big Five test scores), while a negative score indicated underestimation. We describe both underestimation and overestimation of this variable as a mis-estimation.

**Subjective Well-Being (SWB)**. Four different scales were measured and computed into one SWB composite score. For the cognitive component of SWB, Harmony in Life and Satisfaction With Life were measured with the abbreviated three-item versions of the Harmony in Life Scale and the Satisfaction with Life Scale (Kjell & Diener, 2021). Items such as “I am in harmony” and “I am satisfied with my life” were answered on Likert scales ranging from 1 to 7 (*strongly disagree* to *strongly agree*). For the affective component, Positive Affect and Negative Affect were measured using the total score from the Positive and Negative Affect Schedule (Watson et al., 1988). The scale comprises 10 negative (e.g., “Distressed”, “Guilty, and “Upset”) and 10 positive (e.g., “Enthusiastic”, “Inspired” and “Active”) affections on Likert scales ranging from 1 to 5 (*very slightly or not at all* to *extremely*), asking how much participants generally feel the affections. All Cronbach’s alphas and McDonald’s omegas were high, between .89 and .94. It is common to make an SWB composite by summing the SWL and PA scores and then subtracting the NA score, using standardized scores (Romero et al., 2012; Sheldon & Elliot 1999). We did the same thing but used the average of the standardized HIL and SWL scale scores for the cognitive component.

**Self-Insight.** The Self-Insight Scale (Grant et al., 2002) with eight items was used. Examples of items were “I am usually aware of my thoughts” and “I usually know why I feel the way I do”, which were answered on Likert scales ranging from 1 to 6 (*Strongly disagree* to *Strongly agree*). Cronbach’s alpha was 0.90 and McDonald’s omega was 0.93.

**Reliability Check**. A control item was included in the survey: “Please answer the alternative 4 ‘neither agree nor disagree’”. This type of control item has previously demonstrated increased reliability and statistical power in data sets (Oppenheimer et al., 2009). All participants answered correctly on the item.

### Statistical analysis

Hypotheses 1a and b were preliminarily tested using Pearson’s zero-order correlations. We then conducted a hierarchical multiple regression model predicting SWB, from the directed PED in the Big Five^2^, controlling for demographics, Self-Insight, and Big Five scores in a total of four steps. In the first step, Age, Socioeconomic Status, and Sex were entered; in the second step, the PED was entered; in the third step, Self-Insight was entered; and in the final step, the original Big Five test scores were entered, to see if the effects of PED would remain. Hypothesis 2 was tested using a mediation model between PED and SWB, with Self-Insight as the mediator. The average of all Big Five directed PEDs was the independent variable, the Self-Insight measure the mediator, and SWB the dependent variable. As pre-registered, we interpret correlations above 0.3 as strong, between 0.2 and 0.3 as medium, and below 0.2 as small.

All of the study scales and the directed PED had skewness and kurtosis values around 1 or below, which we interpreted as normally distributed. However, all PEDs for the separate Big Five traits had skewness above 1, and kurtosis was above 2 for both extraversion PED and Openness PED, which could be interpreted as too high (Hair et al., 2017). We, therefore, in addition to the initial Pearson correlation, did another Pearson correlation using the log-transformed variables and also Spearman’s rank correlation for these variables. These analyses did not deviate from the initial Pearson correlations presented in the results section. The skewness and kurtosis values, as well as the descriptive statistics of all variables, can be found in *Table S1* in the supplementary material.

The analyses were done in R (R Core Team, 2021) using the RStudio environment (RStudio Team, 2020). The following packages were used: *Hmisc* (Harrell, 2019), *car* (Fox et al., 2020), *psych* (Revelle, 2018), *tidyverse* (Wickham et al., 2019), *lmtest* (Zeileis & Hothorn et al., 2002), *Lavaan* (Rosseel, 2012), *stargazer* (Hlavac, 2018) and *QuantPsych* (Fletcher, 2012). The code is available as open-source.

## Results

*Table S3* in the supplemental material shows how the IPIP-NEO-30 and Self-Perceived personality relate to SWB. *Tables S5-S7* show how the independent variables Self-Perceived Personality, the Big Five, the PED, the directed PED, and the demographic variables sex, age, and socio-economic status were correlated.

### Hypothesis 1a: personality estimation discrepancy (PED) and Subjective Well-Being (SWB)

Pearson’s zero-order correlations for both PED variables (absolute and directed) to SWB and Self-Insight can be found in Table 1, for each Big Five-trait. The first hypothesis (1a) stated that PED would be negatively correlated to SWB. The average absolute PED was not significantly correlated to SWB (*r* = − .11, *p* > .05). Each of the Big Five absolute PEDs showed similar non-significant results. The directed PED was then examined in relation to SWB. The average relative directed PED was on the contrary strongly correlated to SWB (*r* = − .43, *p* < .01). Looking at the directed PED, for each Big Five trait, in Table 1, Extraversion, Emotional Stability, and Conscientiousness had strong significant negative correlations to SWB. The directed PED in Agreeableness had a small significant negative correlation to SWB.

**Table 1:** Correlations between the Study Personality Measurements and Subjective Well-Being (SWB) / Self-Insight (for each of the Big Five Factors)

	Subjective Well-Being (SWB) / Self-Insight					
	E	ES	C	A	O	Mean
absolute PED	− 0.01/-0.06	0.02/ 0.01	− 0.10/ − 0.06	− 0.10/ − 0.14^*^	− 0.04/ − 0.06	− 0.11/ − 0.10
directed PED	**− 0.31**/**-0.17**	**− 0.33**/ **− 0.28**	**− 0.33**/ **− 0.25**	− 0.15^*^/ **− 0.20**	− 0.09/ − 0.10	**− 0.43**/ **− 0.37**

*Note*. IPIP-NEO-30 = Personality Test Scores. PED = Personality Estimation Discrepancy. E = Extraversion. ES = Emotional Stability. C = Conscientiousness. A = Agreeableness. O = Openness. *r* > .17 (bold) was significant at *p* < .01

The first hypothesis (1b) also stated that PED would be negatively correlated to Self-Insight. Seeing how SWB and Self-Insight correlated strongly (*r* = .59), similarly, the average absolute PED was not significantly correlated to Self-Insight (Table 1). Absolute PED in Agreeableness was however negatively correlated to Self-Insight (*r* = − .14, *p* > .05), which means that the higher the absolute value of PED in Agreeableness, the lower the Self-Insight. The remaining PEDs in the Big Five traits were non-significant (*p* > .05). Just as with SWB, the directed average PED was significantly correlated with Self-Insight (*r* = − .37, *p* < .01). All directed PED for the Big Five traits, except for Openness, correlated significantly to Self-Insight (-0.17 < *r* < − .28, *p* < .01). The conclusion of testing the first Hypothesis with correlations is that the relative directed PEDs correlate to SWB and Self-Insight, while the absolute PEDs do not. Further in the analyses, we only utilize the directed PED.

Finally, for Hypothesis 1, we used a four-step multiple hierarchical regression model to examine how much the directed PED in the Big Five traits could predict SWB, controlled for Age, Sex, Socioeconomic Status (in step 1), Self-Insight (step 3; see Table 2) and the Big Five (step 4, not present in Table 2). In the first step, the model including Age, Sex, and Socioeconomic Status performed significantly better than the null model, explaining 13.6% of the variance in SWB (F(3, 293) = 16.5, p < .001). Socioeconomic Status and Age were significant positive predictors. By adding the directed PED in the Big Five traits, the model performed significantly better than the step 1 model, explaining 31.6% of the variance in SWB (F(8, 288) = 18.1, p < .001). The directed PED in Extraversion, Emotional Stability, and Conscientiousness were significant negative predictors. In other words, underestimating one’s personality traits, in general, is associated with high SWB, and particularly, underestimating one’s Extraversion, Emotional Stability, and Conscientiousness is related to high SWB

**Table 2:** Three step Regression Models for Demographics, directed Personality Estimation Discrepancy (PED) and Self-Insight to predict Subjective Well-Being (SWB)

	Dependent variable		
	Subjective Well-Being (SWB)		
	Step 1	Step 2	Step 3
**Demographics**	*β*	*β*	*β*
Socioeconomic Status	0.280^***^	0.231^***^	0.196^***^
Age	0.237^***^	0.157^***^	0.013
Sex **Directed PED**	−0.040	0.030	0.001
Extraversion		−0.253^***^	−0.200^***^
Emotional Stability		−0.259^***^	−0.167^***^
Conscientiousness		−0.180^***^	−0.137^***^
Agreeableness		0.001	0.050
Openness		−0.058	−0.021
Self-Insight			0.450^***^
R2	0.145	0.334	0.484
Adjusted R^2^	0.136	0.316	0.468
Residual Std. Error	2.158 (df = 293)	1.920 (df = 288)	1.693 (df = 287)
F Statistic16.509^***^ (df = 3; 293)18.084^***^ (df = 8; 288)29.895^***^ (df = 9; 287)			

*Note*: N = 297, ^*^p *<* .1; ^**^p *<* .05; ^***^p *<* .01, PED = Personality Estimation Discrepancy

In the third step, adding Self-Insight significantly improved the model, to explain 46.8% of the variance in SWB (F(9, 287) = 29.9, p < .001). Self-Insight was a positive predictor, meaning that participants with a high Self-Insight generally had a high SWB. Further, the significant predictors of the directed PED in the Big Five reduced in strength (but remained significant) after adding Self-Insight. In a final fourth step (not present in Table 2) we added the IPIP-NEO-30 Big Five scores as controls, which increased the explained variance to 67.8% (*F*(14, 282) = 45.5, *p* < .001). All the directed PED Big Five traits dropped to insignificant null results in this step. Thus, the tendency to underestimate one’s personality (directed PED) is indeed related to high SWB, but the relationship is explained by the original IPIP-NEO-30 test-score results. Self-Insight remained a significant predictor of SWB in step 4. The results might suggest that Self-Insight mediates the relationship between the directed PED and SWB

### Hypothesis 2: self-insight mediates PED and SWB

**Fig. 1:**
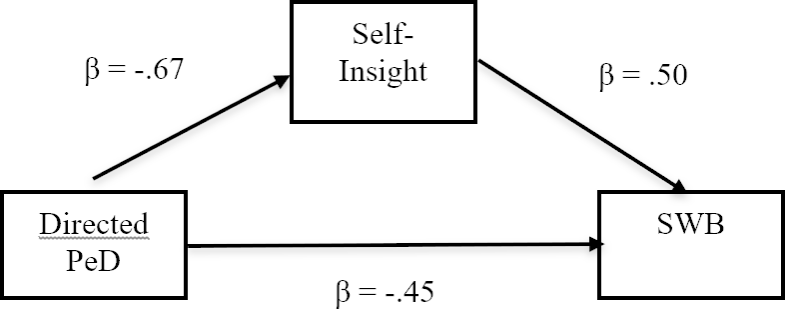
Mediation Model with Self-Insight. (Note: N = 297. Standardized estimates. All effects were significant at p < .001. PED = Personality Estimation Discrepancy. SWB = Subjective Well-Being)

A mediation analysis was performed to test whether Self-Insight would mediate the relationship between the directed PED and SWB. The found significant indirect effect via Self-Insight (B = -0.34, Z = -5.72, p < .001) is depicted in Fig. 1. F(1, 295) = 67.29, R-squared = 0.19, p > .001). The mediating percentage was 42.9% (the non-standardized indirect effect − 0.34 divided by the total effect − 0.79), indicating mediation effects. The conclusion of Hypothesis 2 is that Self-Insight explains a large part of the relationship between directed PED and SWB.

## Discussion

We investigated if the discrepancy between one’s Self-Perceived personality (when provided with trait descriptions) and a regular personality test (i.e., PED) was negatively related to SWB (hypothesis 1a) and Self-Insight (hypothesis 1b). The correlational results implied that the *directed* PED, including the direction (+/-) of mis-estimation, related strongly to SWB and Self-Insight. From the trait perspective, underestimation of Extraversion, Emotional Stability, and Conscientiousness predict both higher SWB and Self-Insight. Conversely, participants who tend to overestimate their personality traits had generally lower SWB and less Self-Insight. The effect of misestimating Extraversion, Emotional Stability, and Conscientiousness on SWB reduced but remained significant when controlling for Self-Insight. However, when controlling for the personality Big Five test scores the relationships disappeared, concluding that Hypothesis 1 was not fully supported. This can be interpreted such that both over-and underestimations of one’s personality largely are driven by the standard Big Five trait scores. In a broader sense, it may be particularly important for people with extreme personalities, especially extremely low scorers on certain traits, to become more aware of their extremities in order to better cope with their behaviors and functioning.

Concerning Hypothesis 2, that Self-Insight is a mediating variable between PED and SWB, we found some support. First, the reduced effect of the directed PED on SWB when adding Self-Insight to the hierarchical regression model (*Table 2*) suggests that Self-Insight mediates the relationship. Indeed, our mediation analysis adds to evidence that Self-Insight has a mediating effect on the relationship. In a wider context, this means that a lower SWB in individuals with a high directed PED is not only driven by the standardized Big Five Personality traits, as suggested in our results of Hypothesis 1, but also in a large part explained by an (mediating) influence of a lack of Self-Insight. Previously, self-reflection has been shown to predict SWB, but only indirectly through Self-Insight (Stein & Grant, 2014). Our results suggest that inaccurate estimations of one’s personality, in the form of overestimating one’s SWB-related Big Five traits, similarly predict low SWB via Self-Insight, possibly by a (lack of) self-reflection mechanism. However, inaccurate estimations of one’s personality in the form of underestimation predict high SWB. Thus, the findings are mixed. Considering the strong influence of Self-Insight on SWB, understanding what mechanisms lead to Self-Insight should be a key question for future positive psychology research.

The focus of the present study was to frame the PED within the Self-Discrepancy Theory (Higgins, 1987). At first glance, the correlational results (*Table 1*) supported that the directed PED can be interpreted according to the theory – that is, the higher the discrepancy, in the form of overestimation, between what one thinks and what one score, the lower the SWB. This finding is in line with findings of previous research regarding Self-Discrepancies predicting SWB (Pavot et al., 1997). Accordingly, the results can be interpreted such that the higher the directed PED, the lower the Self-Insight. This shows that distinguishing within the Actual Self, between implicit and explicit (Self-Perceived) Actual Self, has a theoretical value. Interestingly, underestimation is conversely positively related to SWB and Self-Insight. This contrasts not only with the Self-Discrepancy theory but also with previous findings showing that overestimations of traits and SWB are common and relate to higher SWB (Dufner et al., 2019; Wojcik & Ditto, 2014). These overestimations have often been based on asking individuals how they relate themselves to the average (see Wojcik & Ditto 2014) whereas we computed the misestimations.

It raises the question of whether overestimating one’s extraversion, conscientiousness, and emotional stability are inherent features of a low score on these traits or if these individuals could be more aware of their scores. Thus, a Study that either informs individuals about their scores on these traits or encourages individuals to actively reflect on their personalities could potentially show if a reduction in overestimation of these traits is possible (controlling for Big Five score stability) and if it can increase SWB.

The directed PED - SWB relation comes into question, seeing how the present study results were shown to be fully driven by the Big Five test scores. This driving mechanism is likely further accentuated from a statistical perspective. If a test score is very high, the probability of a lower rating in the Self-Perceived Personality is automatically increased by chance (cf. Regression towards the mean, Nesselroade et al., 1980). The directed PED results may partly be side-effects of scoring high or low in a trait. Nevertheless, the relation is at minimum an inherent feature of personality scores and could be important for therapists and individuals who want to understand humans; individuals who are extreme on a personality trait in the upper end are likely to underestimate their score, and individuals who are extreme in the lower end are likely to overestimate their score.

One could interpret the disappearing relation between PED and SWB after entering the Big Five test scores such that the PED is a weaker one-item measure, and the Big Five test is a stronger multi-item measure, of personality traits. However, we argue that the perceived personality should be seen as the self-perception of one’s personality and not a one-item measure of the Big Five, since comprehensive descriptions of the traits and their labels were provided to the participants. Additionally, the questions asked explicitly for the participants’ self-rating of the traits (e.g., “How extraverted are you?”) rather than asking how much one agrees with a specific statement, which is the case for most Big Five tests, including the IPIP-NEO-30 used in this study.

### Limitations and Future prospects

The limitations of this study should be discussed for future research. First, the study is cross-sectional including self-report measures and thus, causal and objective inferences cannot be made. Further, since the directed PED was shown to be dependent on the Big Five test scores, this raises the question of validity in measuring *perceived* personality traits. In our procedure, we gave our participants not only the names of the traits but also definitions derived from a bipolar single-item measure of the Big Five (Woods & Hampson, 2005). We did this to capture the perceived level of the trait rather than letting the question turn into a one-item measure of the Big Five. However, the descriptions and trait names could have had an influence on both how they perceived themselves as well as the standardized IPIP-NEO-30, which is a potential bias. Perhaps it could be better in the future to measure Self-Perceived Personality via free self-descriptions, similar to the classic method to assess the Actual Self, which could be analyzed quantitatively with modern Natural Language Processing techniques (Kjell et al., 2021). However, this would make a comparison to the Big Five traits, and therefore the calculation of PED, nearly impossible. To measure implicit personality (as we referred to as the Actual Self from a standardized Big Five assessment) more accurately, a multimethod assessment with a composite of peer-reports, self-reports, and maybe even behavioral measures for the Big Five traits could yield a better approach.

We have already mentioned that the mis-estimations may be driven by extreme scores. But what is the reason behind mis-estimations? In future studies, social desirability or availability heuristics could be controlled. Social desirability has previously been shown to influence Big Five scores (Bäckström et al., 2009; Pedregon et al., 2012). Also, humility is proven to be a predictor of SWB (Leary & Guadagno, 2011; Wright et al., 2017), which could be a driving force for mis-estimations of socially desired traits that are related to SWB. Future studies could investigate if individuals scoring extreme on Big Five traits are humbler and have a higher underestimation than those individuals who are less humble, and also use the alternative personality model HEXACO including the honesty-humility factor.

## Conclusions

In this study we defined and introduced a novel distinction within the Self-Discrepancy Theory and a feature of the Big Five personality traits, namely PED, which is the computed difference between the personality a person has according to research and the personality this person perceives he/she has. The results show that the directed PED is strongly related to SWB and Self-Insight. In particular, underestimation of Extraversion, Emotional Stability, and Conscientiousness is related to SWB and Self-Insight. Further analysis reveals that this relation is strongly mediated by Self-Insight but disappears when controlling for the original Big Five trait scores. Nevertheless, directed PED is a feature of personality measurement and happiness studies.

## Electronic supplementary material

Below is the link to the electronic supplementary material.

Supplementary Material 1

Supplementary Material 2

Supplementary Material 3

## Data Availability

Availability of data and material & Code availability can be found here.

## Footnotes

1 Note. Hypothesis 2 was not pre-registered.

2 We did not include absolute PED since their correlations to SWB and Self-Insight were almost exclusively non-significant.
