# Supplementary material for: Know Thyself! Predicting Subjective Well-Being from personality estimation discrepancy and self-insight
Source: Curr Psychol. 2022 Aug 4:1–10. Online ahead of print. doi: 10.1007/s12144-022-03396-1 (PMC9361999; doi:10.1007/s12144-022-03396-1)
Supplement: Supplementary file 1 — Supplementary Material 1 [file 12144_2022_3396_MOESM1_ESM.docx]

**Know Thyself!**

**Predicting Subjective Well-Being from Personality Estimation Discrepancy and Self-Insight - Questionnaire**

***Self-perceived personality***

**Extraversion**

**
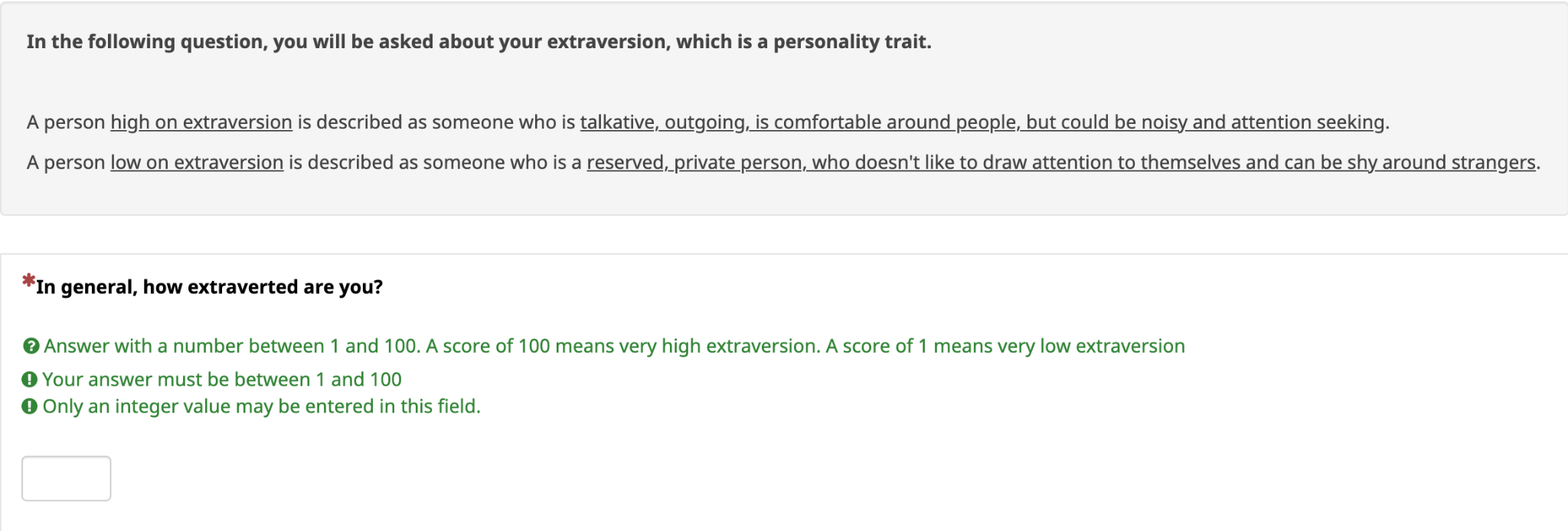
**

**Neuroticism**

**
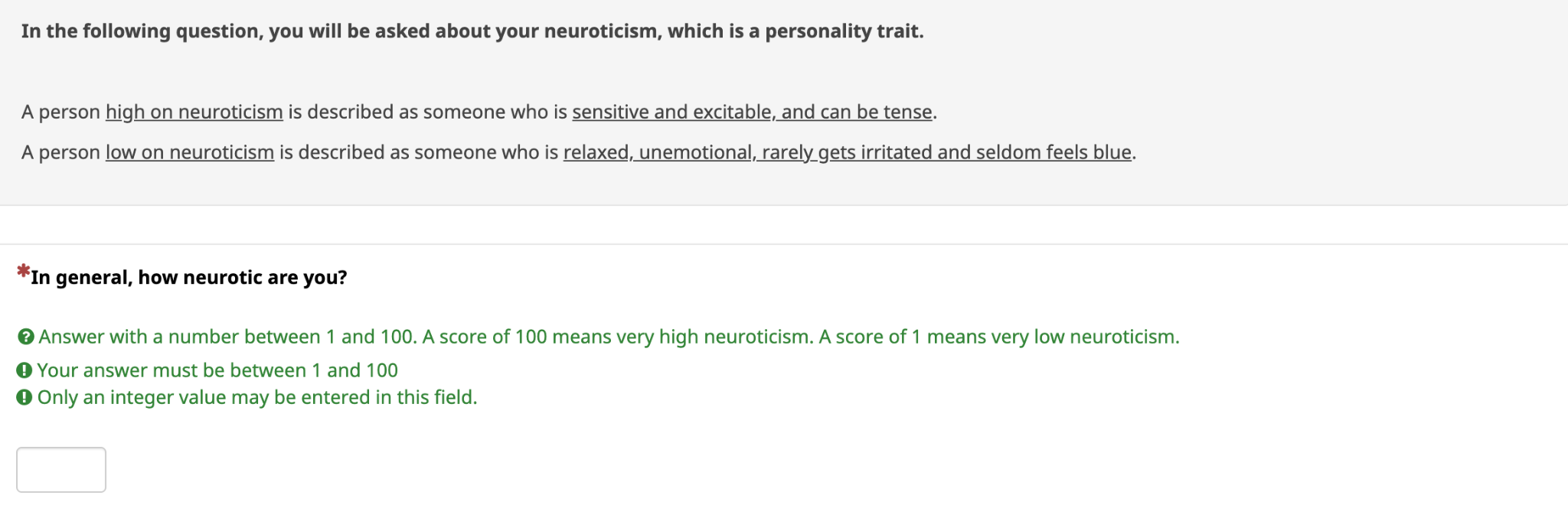
**

**Agreeableness**

**
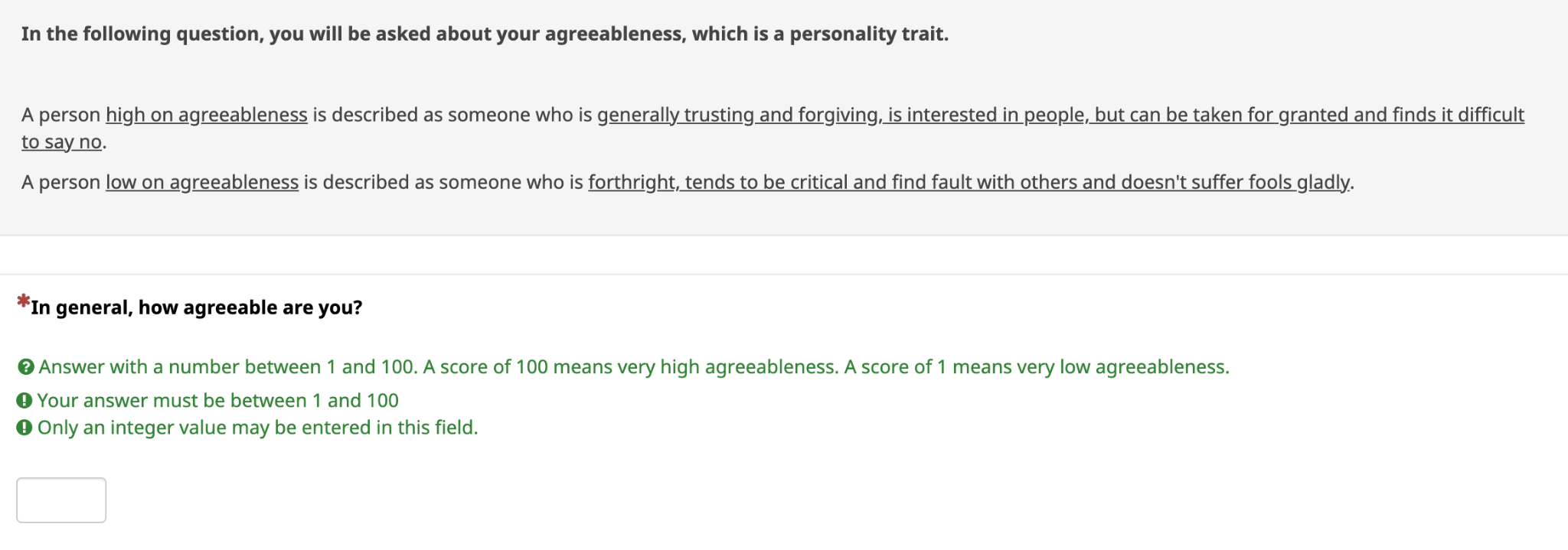
**

**Conscientiousness**

**
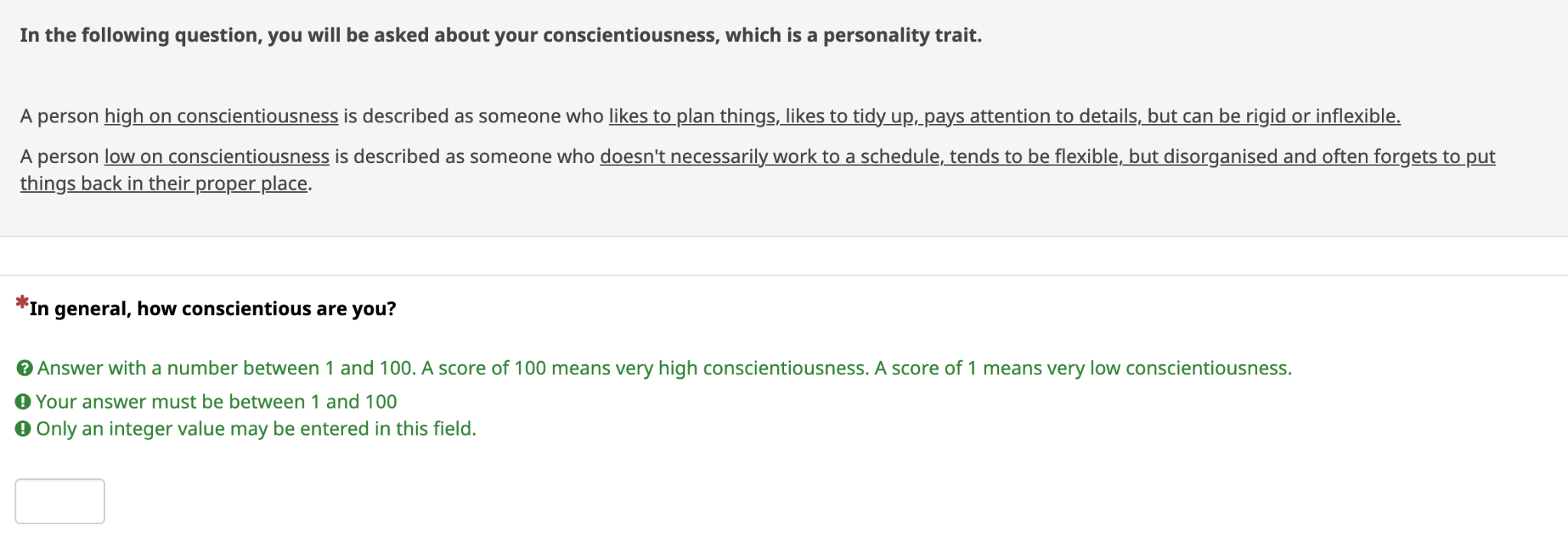
**

**Openness**

**
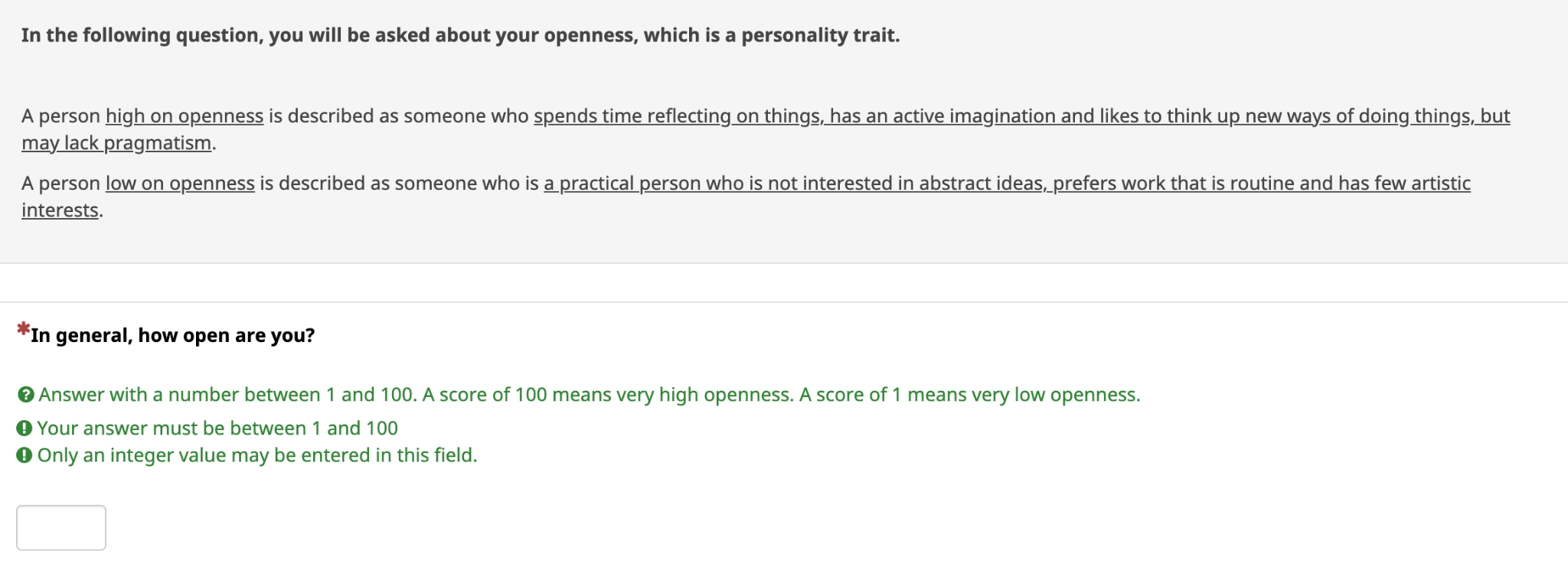
**

***Three item abbreviated Harmony in Life and Satisfaction with Life scales and a control item***

**
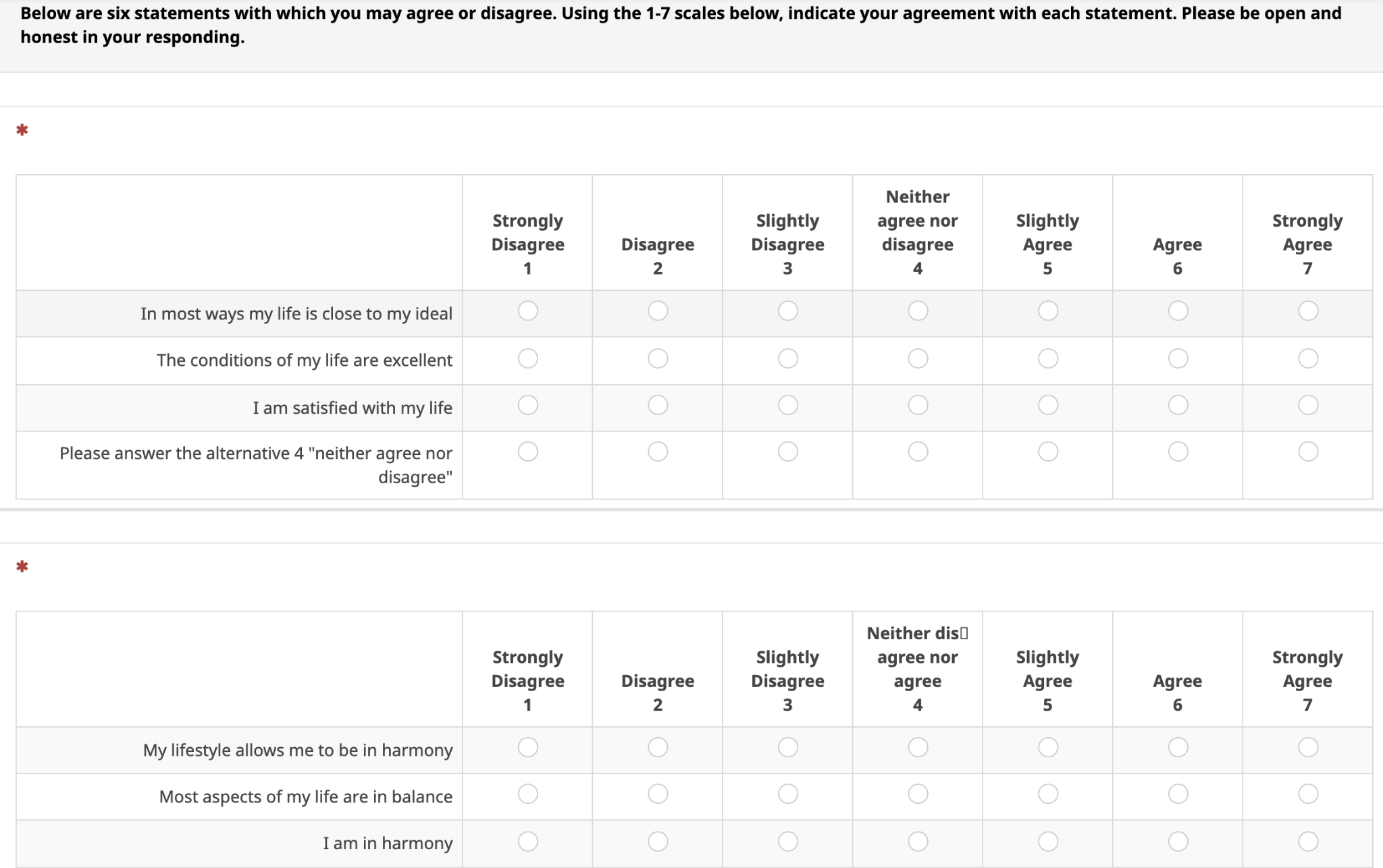
**

***The Positive and Negative Affect schedule***

**
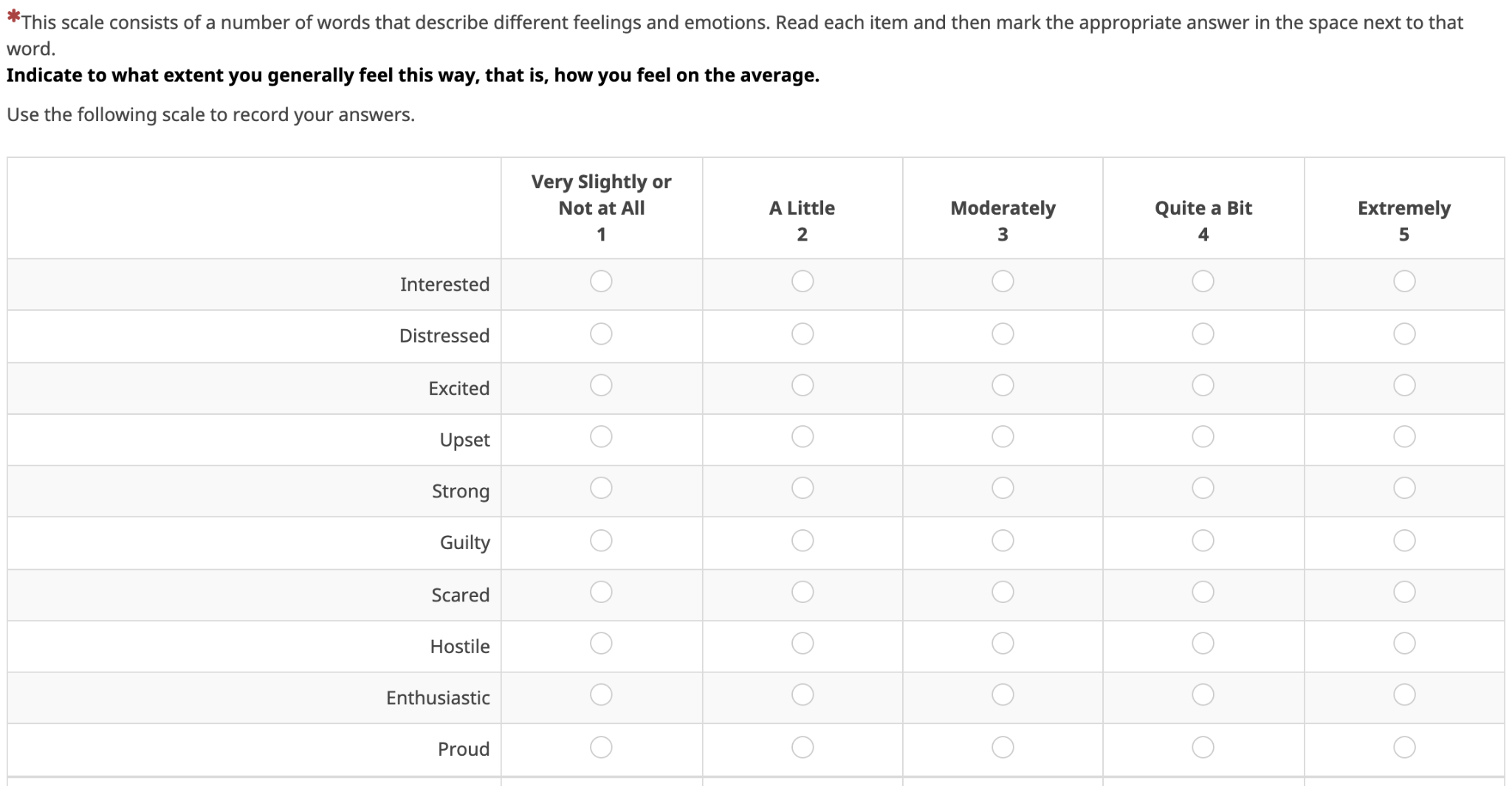
**

**
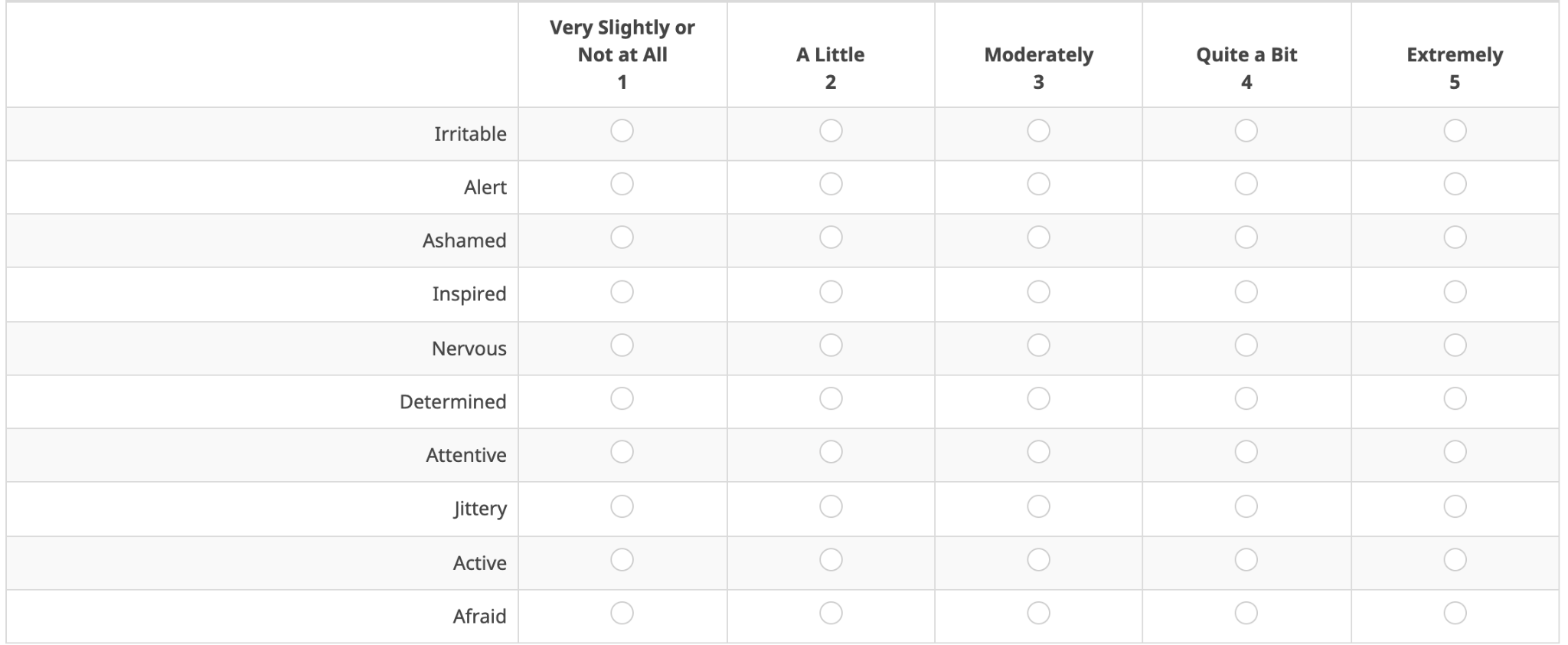
**

***The NEO-IPIP-30 scale***


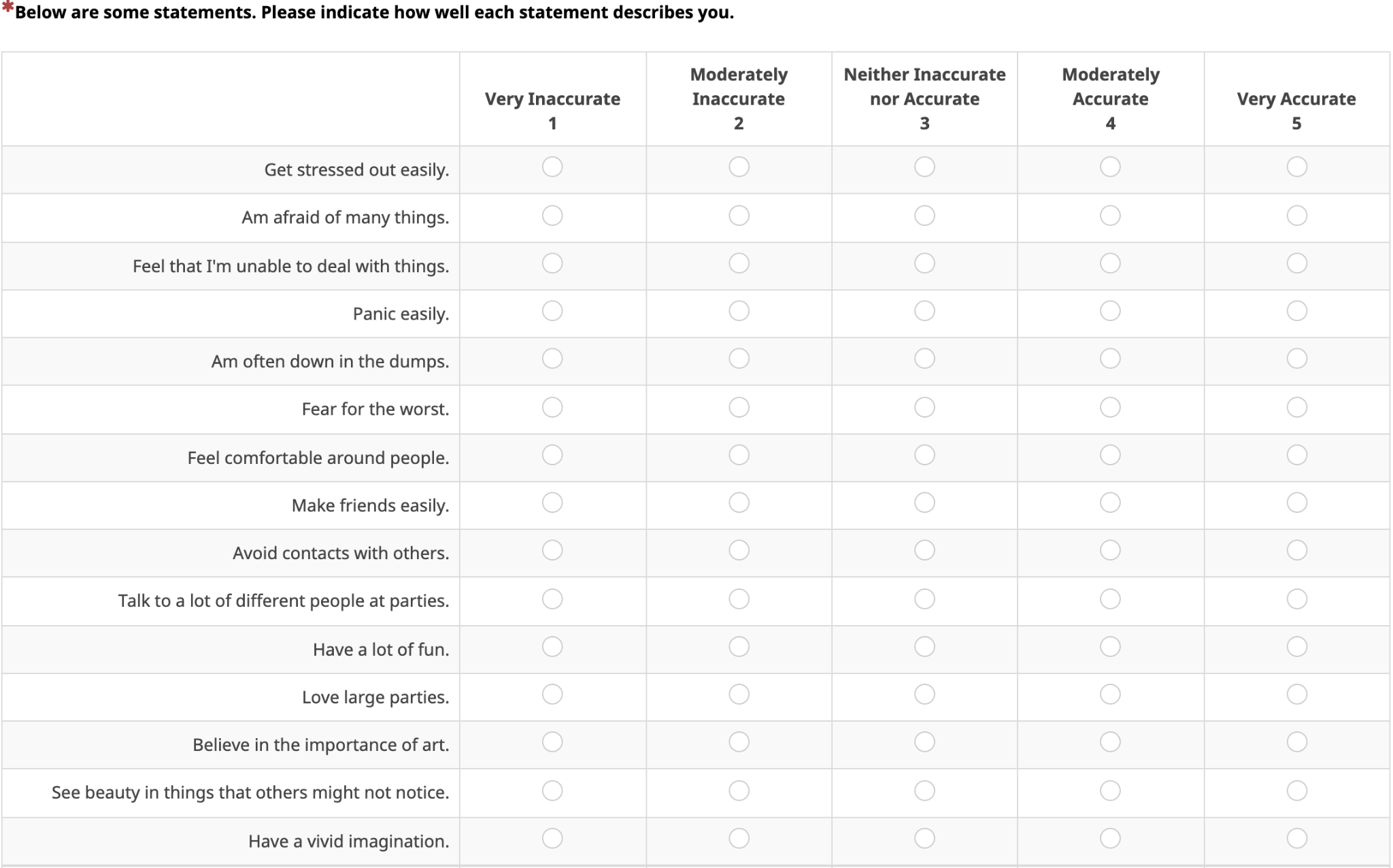


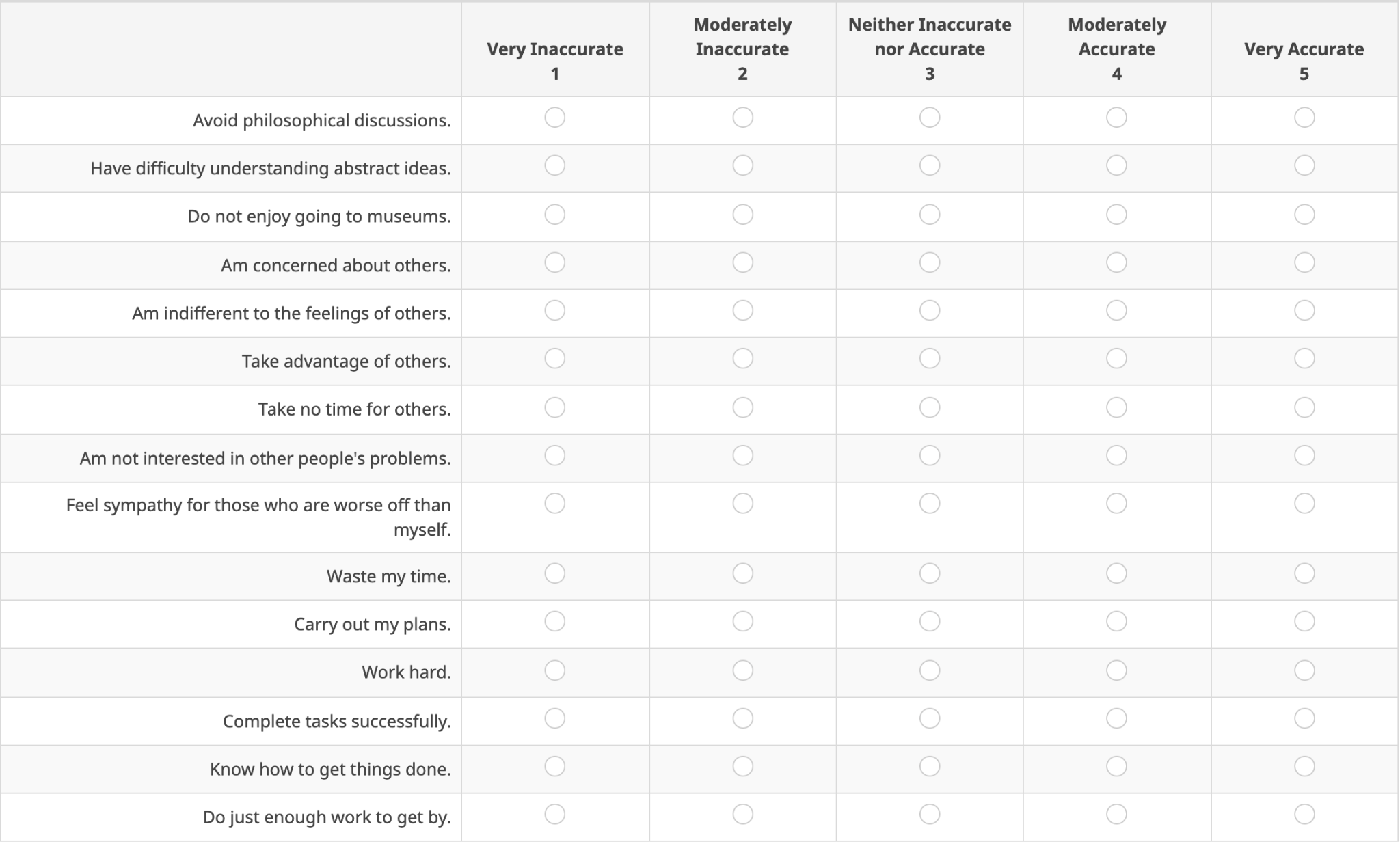


***The Self-Insight scale***


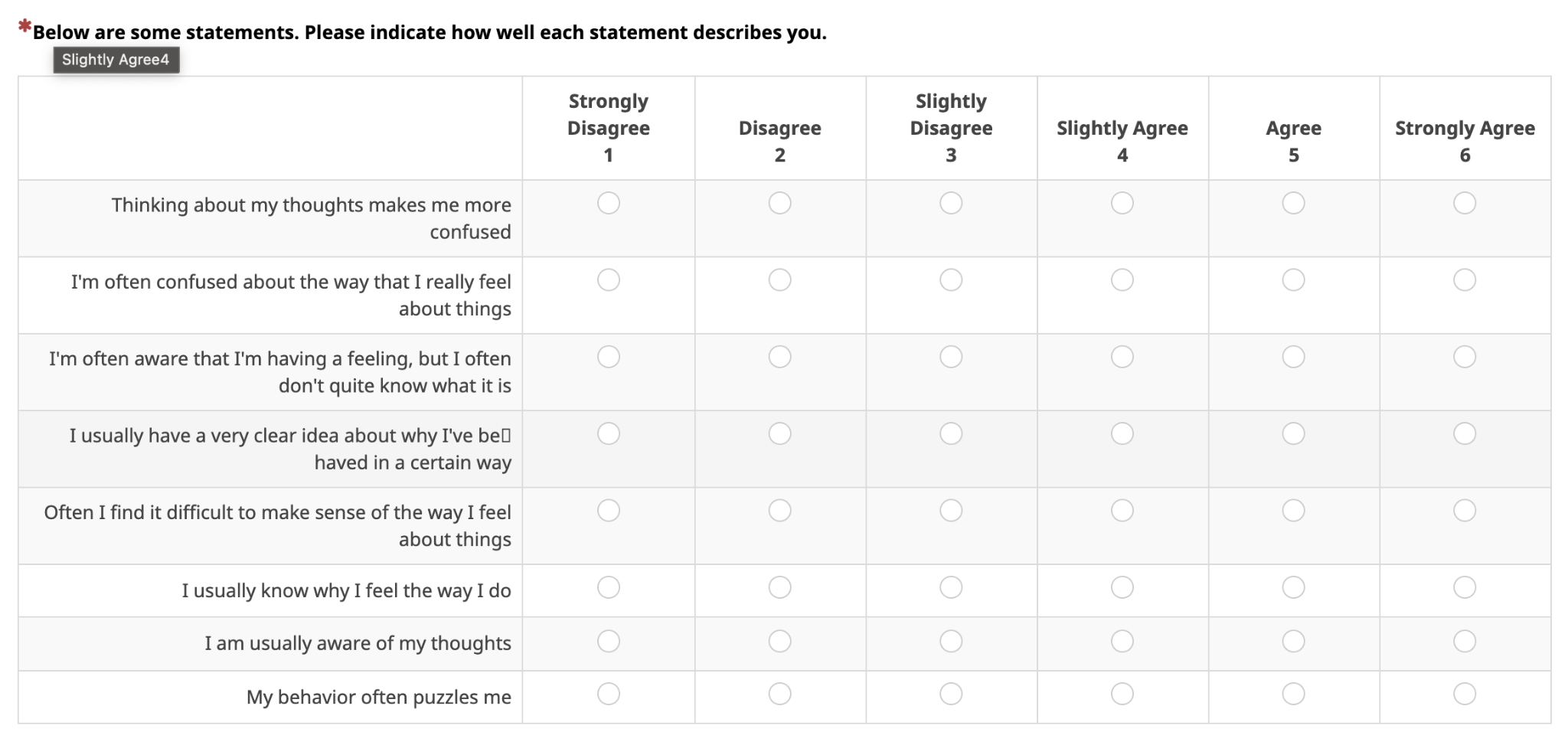


***Participants answered a question concerning Major Life Goals as well that was not a part of the study.***

**
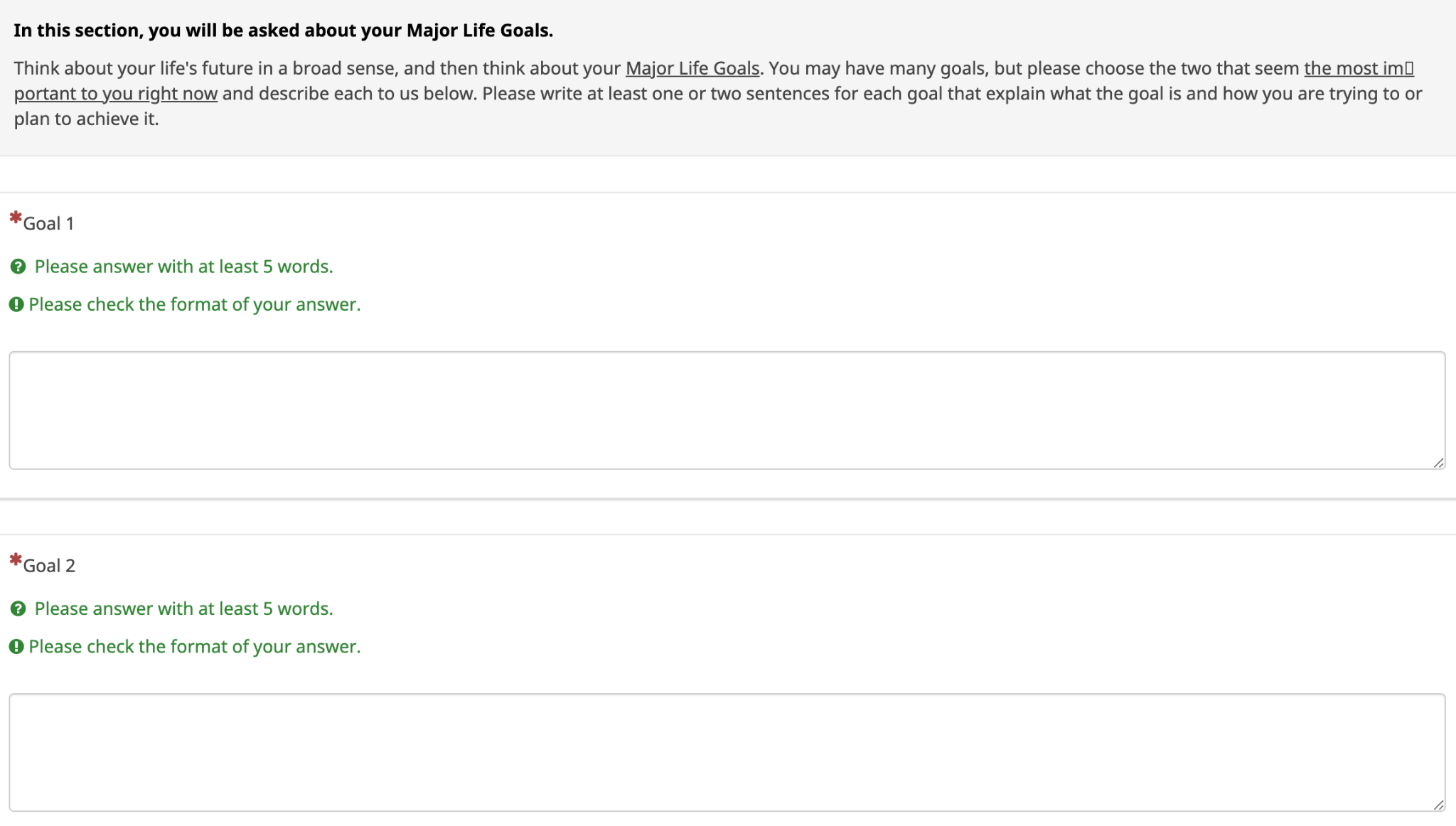
**
