# Supplementary material for: Know Thyself! Predicting Subjective Well-Being from personality estimation discrepancy and self-insight
Source: Curr Psychol. 2022 Aug 4:1–10. Online ahead of print. doi: 10.1007/s12144-022-03396-1 (PMC9361999; doi:10.1007/s12144-022-03396-1)
Supplement: Supplementary file 3 — Supplementary Material 3 [file 12144_2022_3396_MOESM3_ESM.docx]

**Know Thyself: the Role of Personality Discrepancy in predicting Self-Insight and Subjective Well-Being**

# Supplemental Material

[August Nilsson](mailto:august.nilsson@psy.lu.se)^1^, [Kira Friedrichs](mailto:friedrichs.kira@gmail.com)^1^  and [Petri Kajonius](mailto:Petri.Kajonis@psy.lu.se)^1^

^1^Department of Psychology, Lund University, Sweden

**Corresponding author:** August Nilsson. [august.nilsson@psy.lu.se](mailto:august.nilsson@psy.lu.se)

#

**Table S1.** *Descriptive statistics for the numerical variables.*

Variables Mean *SD* Skew Kurtosis Scale range

Dependent variables

SWB .00 2.32 -.43 -.09 1-100

HILS-3 12.84 4.06 -.35 -.73 3-18

SWLS-3 12.60 4,35 -.35 -.77 3-18

PA scale 30.93 7.21 -.06 -.52 10-50

NA scale 19.85 8.14 1.02 .64 10-50

Self-Insight 33.12 7.96 -.17 -.43 8-48

IPIP-NEO-30

Extraversion 18.55 5.11 .01 -.88 6-30

Emotional Stability 19.78 6.40 -.15 -.96 6-30

Conscientiousness 21.92 4.23 -.30 -.17 6-30

Agreeableness 25.09 3.67 -.83 .56 6-30

Openness 22.46 4.49 -.53 -.03 6-30

Self-Perceived Personality

Extraversion 43.06 22.19 .06 -.87 1-100

Emotional Stability 46.40 25.12 .21 -.93 1-100

Conscientiousness 67.18 22.65 -.58 -.35 1-100

Agreeableness 62.30 22.36 -.58 -.41 1-100

Openness 63.30 22.24 -.53 -.12 1-100

Personality Discrepancy Min-Max

Extraversion .66 .53 1.35 2.38 0.01 - 2.92

Neuroticism .69 .55 1.10 .84 0.01 - 2.79

Conscientiousness .86 .70 1.06 .68 0.01 - 3.51

Agreeableness .91 .79 1.30 1.62 0.00 - 3.99

Openness .77 .63 1.27 2.00 0.00 - 3.75

Average .78 .32 .87 .76 0.02 - 1.95

Directed Personality Discrepancy

Extraversion .00 .84 -.30 -63 -2.92 - 2.53

Emotional Stability .00 .89 .11 .18 -2.45 - 2.79

Conscientiousness .00 1.11 -.07 .18 -3.11 - 3.51

Agreeableness .00 1.20 .02 .71 -3.89 - 3.99

Openness .00 .99 .23 .57 -2.74 - 3.75

Average .00 .55 -.08 -.04 -1.43 - 1.60

*Note*. N = 297. SWB = Subjective Well-Being, HILS-3 = Harmony in Life Scale three item version, SWLS-3 = Satisfaction with Life Scale three item version, PA = Positive Affect, NA = Negative Affect, IPIP-NEO-30 = International Personality Item Pool-Neuroticism Extraversion Openness 30 item version.

**Table S2.**

*Correlations between SWB scales scores, Self-Insight scores and demographic variables..*

Variables 1 2 3 4 5 6

1. SWB

2. HILS-3 .83**

3. SWLS-3 .77** .78**

4. Positive Affect scale .76** .49** .45**

5. Negative Affect scale -.77** -.55** -.44** -.29**

6. Self-Insight .59** .45** .37** .36** -.60**

*Note*. N = 295. All *p* < .001 (2-tailed). SWB = Subjective Well-Being composite of remaining SWB variables. HILS-3 = Harmony in Life Scale three item version, SWLS-3 = Satisfaction with Life Scale three item version

**Table S3.**

Correlations between the Study Personality Measurements and Subjective Well-Being (SWB) / Self-Insight (for each of the Big Five Factors)

|  | Subjective Well-Being (SWB) / Self-Insight | | | | | |
| --- | --- | --- | --- | --- | --- | --- |
|  | E | ES | C | A | O | Mean |
| Self-Perceived Personality | **.32**/**.18** | **.42**/**.41** | .15/ .16 | .08/ -.03 | .05/ -.01 | **.45**/**.32** |
| IPIP-NEO-30 | **.59**/**.33** | **.72**/ **.66** | **.51**/ **.44** | **.26**/ **.21** | .13/.10 | **.75**/**.59** |
|  |  |  |  |  |  |  |

*Note*. IPIP-NEO-30 = Personality Test Scores. E = Extraversion. ES = Emotional Stability. C = Conscientiousness. A = Agreeableness. O = Openness. *r* > .173 (bold) was significant at *p* < .01

**Table S4.**

*Regression models of the directed PED for each trait and the Big Five on the SWB measures and Self-Insight.*

Variables HILS-3 SWLS-3 PA NA^L^

β β β β

Step 1

directed PED

Extraversion -.27** -.22** -.22** .13*

Emotional Stability -.24** -.21** -.20** .24**

Conscientiousness -.13* -.17** -.14* .25**

Agreeableness -.08 .01 .02 -.03

Openness .09 .07 -.01 -.08

*R^2^* .17 .16 .14 .19

Adjusted *R^2^*  .16** .15** .12** .17**

F (5, 291) 12.31 11.22 9.14 12.25

IPIP-NEO-30

Extraversion .29** .30** .36** -.00

Emotional Stability .34** .25** .19** -.69**

Conscientiousness .15** .13* .31** -.14**

Agreeableness .02 .05 -.02 -.05

Openness -.04 -.02 .11* -.06

*R^2^* .37 .29 .45 .57

Adjusted *R^2^*  .36** .28** .44** .56**

F (5, 291) 34.06 24.34 48.05 71.89

*Note*. N = 297. * *p* < .05; ** *p* < .01. SWB = Subjective Well-Being composite score,
HILS-3 = Harmony in Life Scale three item version, SWLS-3 = Satisfaction with Life Scale three item version, PA = Positive Affect, NA = Negative Affect, IPIP-NEO-30 = International Personality Item Pool-Neuroticism Extraversion Openness 30 item version, ^L^=Log transformed dependent variable.

**Table S5.**

*Correlations between Study Variables.*

Variables 1 2 3 4 5 6 7 8 9 10 11 12 13 14

Directed Personality Discrepancy

1. Extraversion
2. Emotional Stability .04
3. Conscientiousness .17** .17**
4. Agreeableness .16** .16** .22**
5. Openness .01 .03 .06 .07
6. Average .47** .49** .63** .66** .43**

IPIP-NEO-30

1. Extraversion -.42** -.30** -.21** -.17** -.03 -.39**
2. Emotional Stability -.20** -.44** -.28** -.10 -.06 -.39** .44**
3. Conscientiousness -.24** -.24** -.55** -.27** -.11 -.53** .27** .36**
4. Agreeableness -.20** -.21** -.21** -.60** -.05 -.50** .32** .10 .32**
5. Openness .01 -.10 -.04 -.23** -.50** -.33** .21** .06 .06 .21**

Demographics

1. Sex^a^ .11 .09 .10 .16** .02 .18** -.08 .11 -.06 -.25** .02
2. Age -.03 -.12* -.23** -.09 .00 -.18** .05 .33** .33** .20** -.01 .03
3. Socioeconomic status -.07 -.07 -.10 -.08 -.04 -.14* .22** .22** .07 .07 .02 -.02 .07

*Note*. N = 297. * *p* < .05; ** *p* < .01 (2-tailed). IPIP-NEO-30 = International Personality Item Pool-Neuroticism Extraversion Openness 30 item version, ^a^ 2=male, 1=female

**Table S6.**

*Correlations between Personality Discrepancy scores and other independent and demographic variables.*

Variables 1 2 3 4 5 6

Personality Discrepancy

1. Extraversion
2. Emotional Stability .02
3. Conscientiousness -.01 .08
4. Agreeableness .05 .03 .10
5. Openness .07 .11 .12* .03
6. Average .39** -.44** -.56** -.55** .50**

IPIP-NEO-30

Extraversion .07 .01 -.08 -.08 .00 -.05

Emotional Stability -.01 -.05 -.06 -.05 -.11 -.11*

Conscientiousness .13* .03 -.13* -.07 .01 -.03

Agreeableness -.01 .03 .00 -.25** -.17** -.18**

Openness -.03 -.01 -.02 -.03 -.26** -.14*

Directed Personality Discrepancy

Extraversion -.11* -.04 -.06 -.05 -.01 -.11

Emotional Stability .04 .05 .00 .04 .10 .09

Conscientiousness -.04 .02 -.04 .05 .02 .01

Agreeableness -.04 -.04 -.01 -.01 .04 -.02

Openness .11 .03 -.05 -.05 .11 -.04

Average -.02 .01 -.06 -.01 .09 .01

Self-Perceived Personality

Extraversion -.03 -.02 -.13* -.12* .01 -.14*

Emotional Stability .03 -.01 -.06 -.01 -.02 -.04

Conscientiousness .08 .05 -.17** .02 .03 -.03

Agreeableness -.06 -.02 .00 -.26** -.12* -.20**

Openness .07 .02 -.07 -.08 -.15* -.10

Demographics

Sex^a^ -.06 -.06 -.03 -.08 -.03 -.03

Age .05 -.04 -.10 -.28 .13* -.18**

Socioeconomic status -.04 -.02 -.10 -.01 .10 -.03

*Note*. N = 297 * *p* < .05; ** *p* < .01 (2-tailed). ^a^ 2=male, 1=female.

**Table S7.**

*Correlations between Self-Perceived Personality scores and other independent and demographic variables.*

Variables 1 2 3 4 5

Self-Perceived Personality

1. Extraversion
2. Emotional Stability .03
3. Conscientiousness .00 .01
4. Agreeableness .12* -.03 .05
5. Openness .20** -.07 -.03 -.02

IPIP-NEO-30

Extraversion .64** -.17** .04 .12* .18**

Emotional Stability -.26** .61** -.05 .02 .00

Conscientiousness .07 -.15* .39** -.01 -.05

Agreeableness .16** .08 .09 .27** .16**

Openness .21** .03 .01 -.06 .51**

Directed Personality Discrepancy

Extraversion .42** .17** -.05 .00 .02

Emotional Stability .27** .44** .05 .01 .07

Conscientiousness -.06 .12* .55** .05 .01

Agreeableness -.03 -.04 .03 .60** -.15**

Openness -.02 .01 .04 .04 .50**

Average .00 .05 .16** .30** .10

Demographics

Sex^a^ .01 -.19** .05 -.06 -.02

Age .02 -.22** .07 .08 -.01

Socioeconomic Status .16** -.15** -.04 -.03 .04

*Note*. N = 297 * *p* < .05; ** *p* < .01 (2-tailed). ^a^ 2=male, 1=female.
